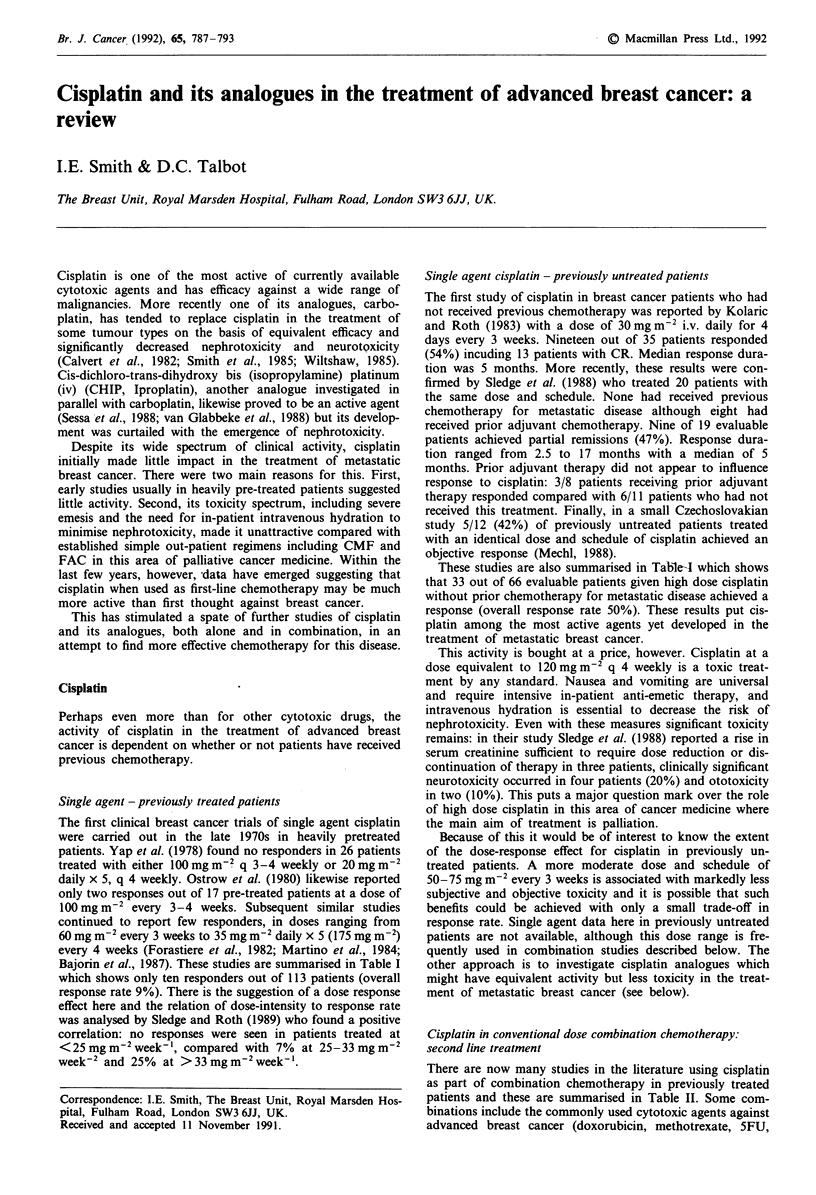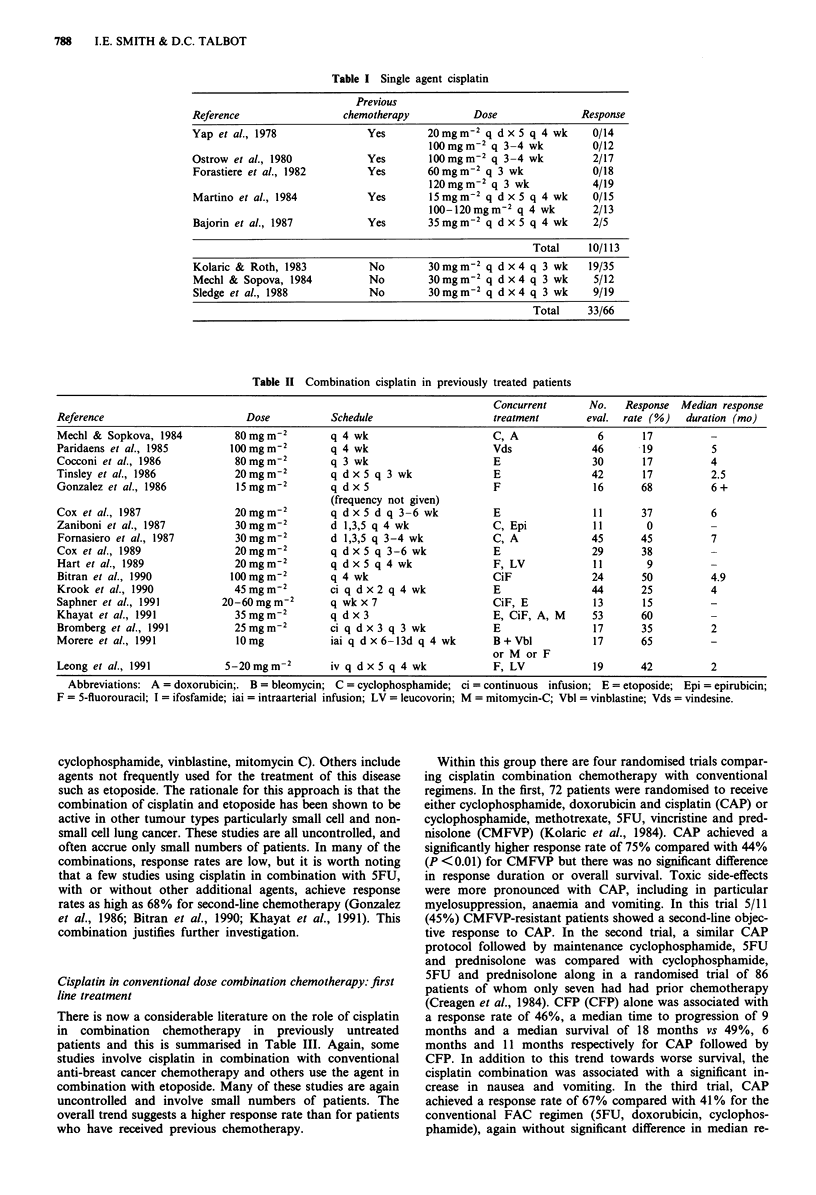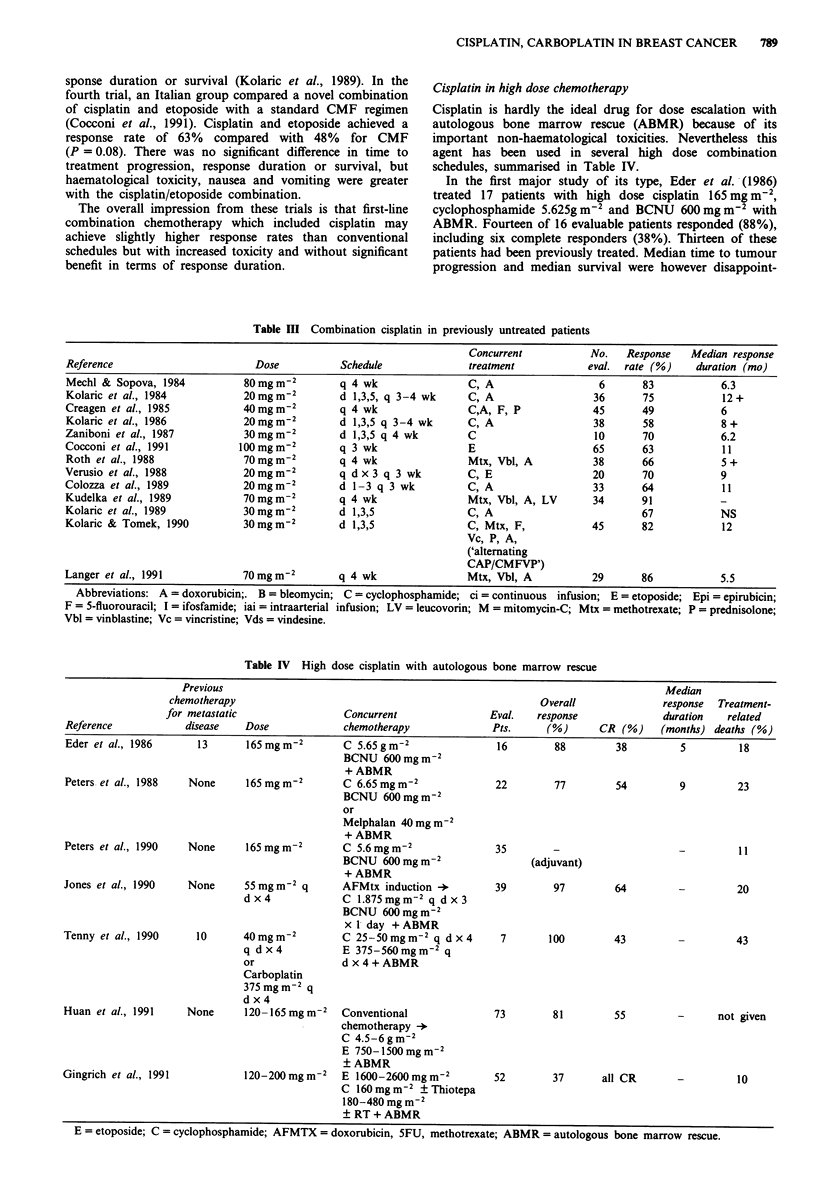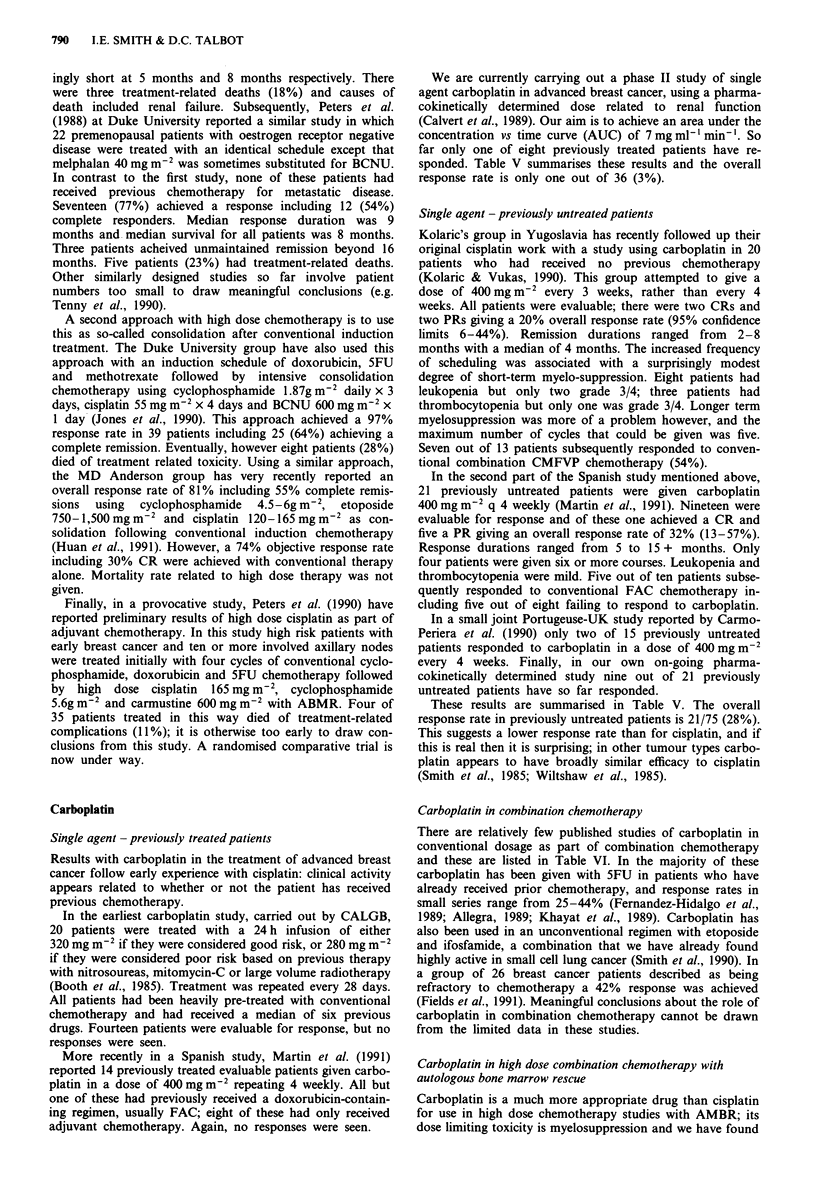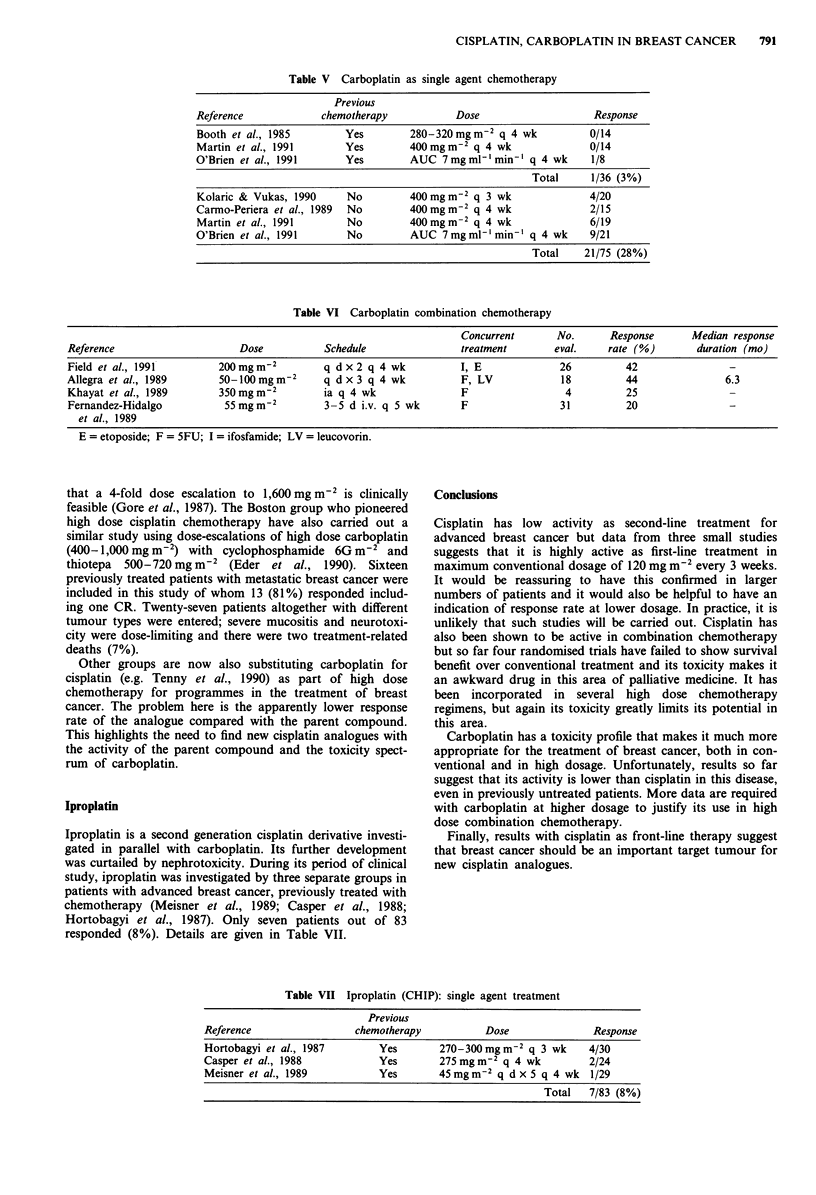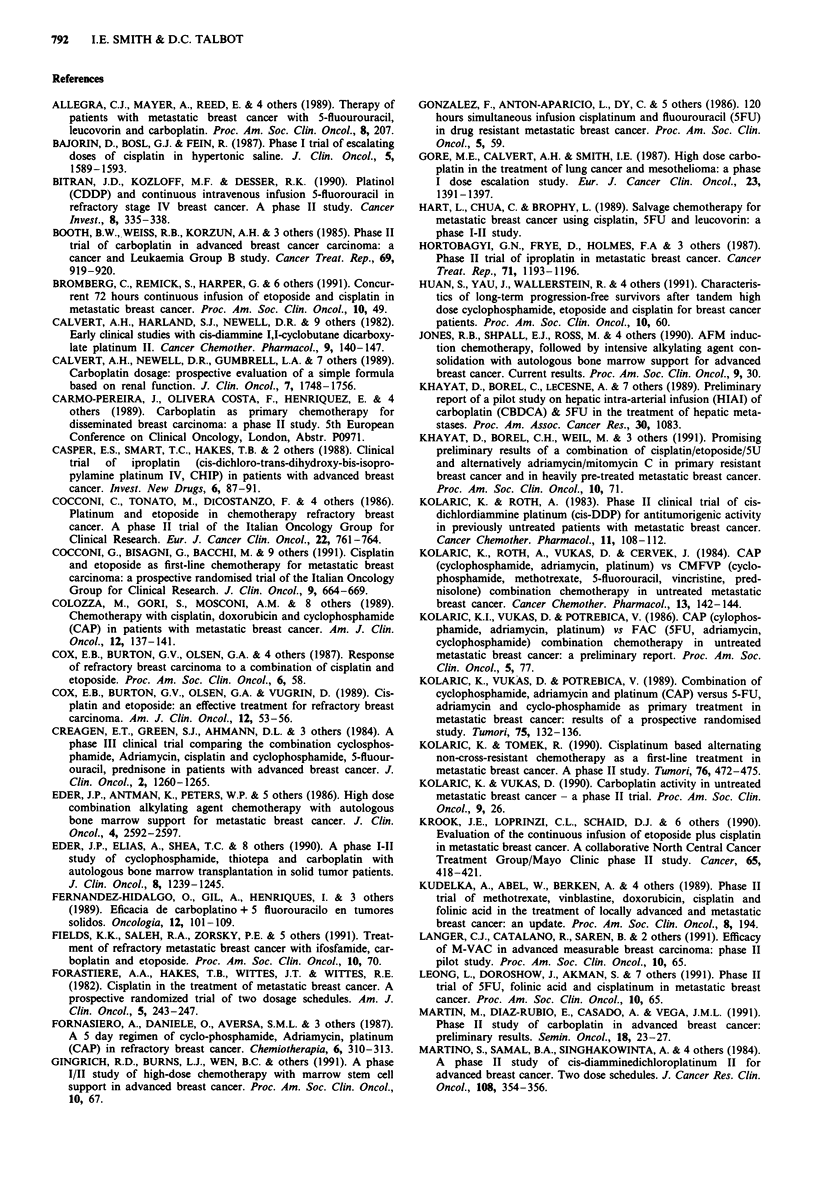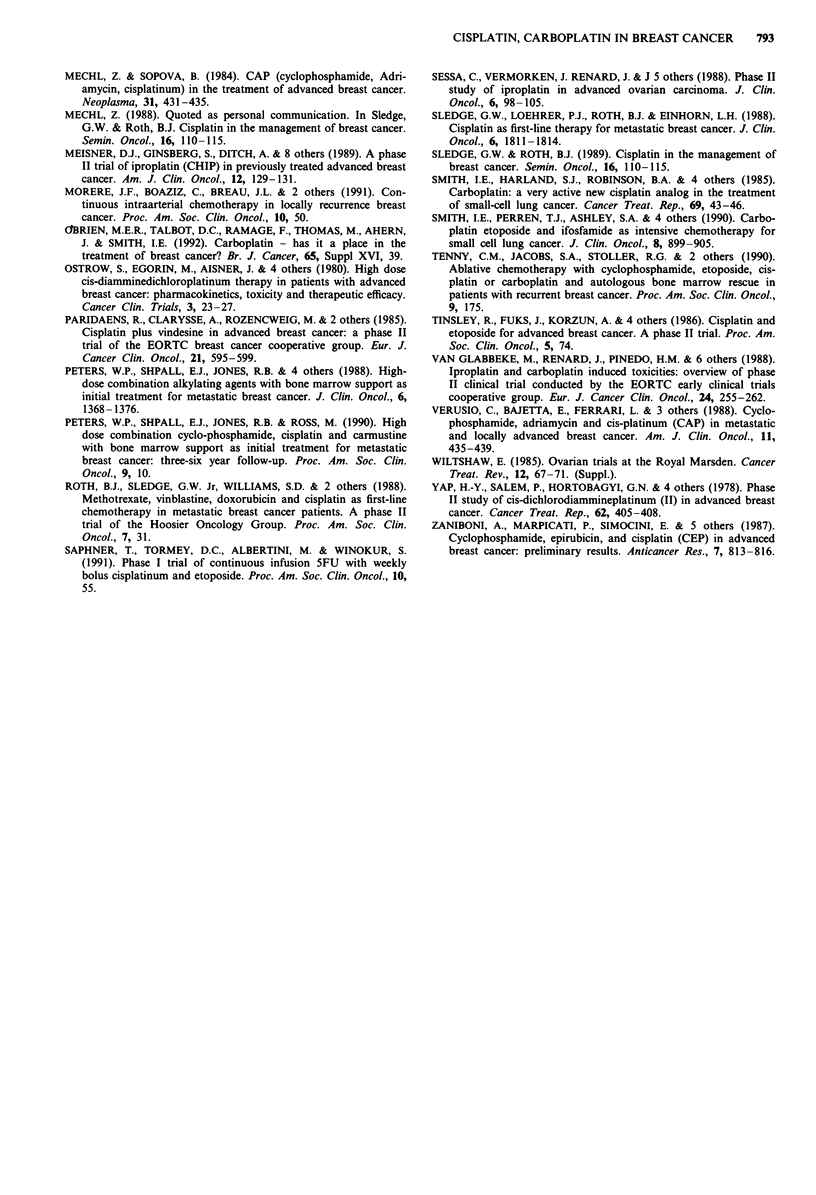# Cisplatin and its analogues in the treatment of advanced breast cancer: a review.

**DOI:** 10.1038/bjc.1992.169

**Published:** 1992-06

**Authors:** I. E. Smith, D. C. Talbot

**Affiliations:** Breast Unit, Royal Marsden Hospital, London, UK.


Br. J. Cancer (1992), 65, 787–793 © Macmillan Press Ltd., 1992

### Cisplatin and its analogues in the treatment of advanced breast cancer: a review

I.E. Smith & D.C. Talbot

*The Breast Unit, Royal Marsden Hospital, Fulham Road, London SW3 6JJ, UK.*

Cisplatin is one of the most active of currently available cytotoxic agents and has efficacy against a wide range of malignancies. More recently one of its analogues, carboplatin, has tended to replace cisplatin in the treatment of some tumour types on the basis of equivalent efficacy and significantly decreased nephrotoxicity and neurotoxicity (Calvert *et al.*, 1982; Smith *et al.*, 1985; Wiltshaw, 1985). Cis-dichloro-trans-dihydroxy bis (isopropylamine) platinum (iv) (CHIP, Iproplatin), another analogue investigated in parallel with carboplatin, likewise proved to be an active agent (Sessa *et al.*, 1988; van Glabbeke *et al.*, 1988) but its development was curtailed with the emergence of nephrotoxicity.

Despite its wide spectrum of clinical activity, cisplatin initially made little impact in the treatment of metastatic breast cancer. There were two main reasons for this. First, early studies usually in heavily pre-treated patients suggested little activity. Second, its toxicity spectrum, including severe emesis and the need for in-patient intravenous hydration to minimise nephrotoxicity, made it unattractive compared with established simple out-patient regimens including CMF and FAC in this area of palliative cancer medicine. Within the last few years, however, data have emerged suggesting that cisplatin when used as first-line chemotherapy may be much more active than first thought against breast cancer.

This has stimulated a spate of further studies of cisplatin and its analogues, both alone and in combination, in an attempt to find more effective chemotherapy for this disease.

### Cisplatin

Perhaps even more than for other cytotoxic drugs, the activity of cisplatin in the treatment of advanced breast cancer is dependent on whether or not patients have received previous chemotherapy.

### *Single agent – previously treated patients*

The first clinical breast cancer trials of single agent cisplatin were carried out in the late 1970s in heavily pretreated patients. Yap *et al.* (1978) found no responders in 26 patients treated with either 100 mg m$^{-2}$ q 3–4 weekly or 20 mg m$^{-2}$ daily × 5, q 4 weekly. Ostrow *et al.* (1980) likewise reported only two responses out of 17 pre-treated patients at a dose of 100 mg m$^{-2}$ every 3–4 weeks. Subsequent similar studies continued to report few responders, in doses ranging from 60 mg m$^{-2}$ every 3 weeks to 35 mg m$^{-2}$ daily × 5 (175 mg m$^{-2}$) every 4 weeks (Forastiere *et al.*, 1982; Martino *et al.*, 1984; Bajorin *et al.*, 1987). These studies are summarised in Table I which shows only ten responders out of 113 patients (overall response rate 9%). There is the suggestion of a dose response effect here and the relation of dose-intensity to response rate was analysed by Sledge and Roth (1989) who found a positive correlation: no responses were seen in patients treated at $<$25 mg m$^{-2}$ week$^{-1}$, compared with 7% at 25–33 mg m$^{-2}$ week$^{-2}$ and 25% at $>$33 mg m$^{-2}$ week$^{-1}$.

Correspondence: I.E. Smith, The Breast Unit, Royal Marsden Hospital, Fulham Road, London SW3 6JJ, UK.
Received and accepted 11 November 1991.

### *Single agent cisplatin – previously untreated patients*

The first study of cisplatin in breast cancer patients who had not received previous chemotherapy was reported by Kolaric and Roth (1983) with a dose of 30 mg m$^{-2}$ i.v. daily for 4 days every 3 weeks. Nineteen out of 35 patients responded (54%) incuding 13 patients with CR. Median response duration was 5 months. More recently, these results were confirmed by Sledge *et al.* (1988) who treated 20 patients with the same dose and schedule. None had received previous chemotherapy for metastatic disease although eight had received prior adjuvant chemotherapy. Nine of 19 evaluable patients achieved partial remissions (47%). Response duration ranged from 2.5 to 17 months with a median of 5 months. Prior adjuvant therapy did not appear to influence response to cisplatin: 3/8 patients receiving prior adjuvant therapy responded compared with 6/11 patients who had not received this treatment. Finally, in a small Czechoslovakian study 5/12 (42%) of previously untreated patients treated with an identical dose and schedule of cisplatin achieved an objective response (Mechl, 1988).

These studies are also summarised in Table I which shows that 33 out of 66 evaluable patients given high dose cisplatin without prior chemotherapy for metastatic disease achieved a response (overall response rate 50%). These results put cisplatin among the most active agents yet developed in the treatment of metastatic breast cancer.

This activity is bought at a price, however. Cisplatin at a dose equivalent to 120 mg m$^{-2}$ q 4 weekly is a toxic treatment by any standard. Nausea and vomiting are universal and require intensive in-patient anti-emetic therapy, and intravenous hydration is essential to decrease the risk of nephrotoxicity. Even with these measures significant toxicity remains: in their study Sledge *et al.* (1988) reported a rise in serum creatinine sufficient to require dose reduction or discontinuation of therapy in three patients, clinically significant neurotoxicity occurred in four patients (20%) and ototoxicity in two (10%). This puts a major question mark over the role of high dose cisplatin in this area of cancer medicine where the main aim of treatment is palliation.

Because of this it would be of interest to know the extent of the dose-response effect for cisplatin in previously untreated patients. A more moderate dose and schedule of 50–75 mg m$^{-2}$ every 3 weeks is associated with markedly less subjective and objective toxicity and it is possible that such benefits could be achieved with only a small trade-off in response rate. Single agent data here in previously untreated patients are not available, although this dose range is frequently used in combination studies described below. The other approach is to investigate cisplatin analogues which might have equivalent activity but less toxicity in the treatment of metastatic breast cancer (see below).

### *Cisplatin in conventional dose combination chemotherapy: second line treatment*

There are now many studies in the literature using cisplatin as part of combination chemotherapy in previously treated patients and these are summarised in Table II. Some combinations include the commonly used cytotoxic agents against advanced breast cancer (doxorubicin, methotrexate, 5FU,

**Table I** Single agent cisplatin

| *Reference* | *Previous chemotherapy* | *Dose* | *Response* |
|---|---|---|---|
| Yap *et al.*, 1978 | Yes | 20 mg m$^{-2}$ q d × 5 q 4 wk | 0/14 |
| | | 100 mg m$^{-2}$ q 3–4 wk | 0/12 |
| Ostrow *et al.*, 1980 | Yes | 100 mg m$^{-2}$ q 3–4 wk | 2/17 |
| Forastiere *et al.*, 1982 | Yes | 60 mg m$^{-2}$ q 3 wk | 0/18 |
| | | 120 mg m$^{-2}$ q 3 wk | 4/19 |
| Martino *et al.*, 1984 | Yes | 15 mg m$^{-2}$ q d × 5 q 4 wk | 0/15 |
| | | 100–120 mg m$^{-2}$ q 4 wk | 2/13 |
| Bajorin *et al.*, 1987 | Yes | 35 mg m$^{-2}$ q d × 5 q 4 wk | 2/5 |
| | | Total | 10/113 |
| Kolaric & Roth, 1983 | No | 30 mg m$^{-2}$ q d × 4 q 3 wk | 19/35 |
| Mechl & Sopova, 1984 | No | 30 mg m$^{-2}$ q d × 4 q 3 wk | 5/12 |
| Sledge *et al.*, 1988 | No | 30 mg m$^{-2}$ q d × 4 q 3 wk | 9/19 |
| | | Total | 33/66 |

**Table II** Combination cisplatin in previously treated patients

| *Reference* | *Dose* | *Schedule* | *Concurrent treatment* | *No. eval.* | *Response rate (%)* | *Median response duration (mo)* |
|---|---|---|---|---|---|---|
| Mechl & Sopkova, 1984 | 80 mg m$^{-2}$ | q 4 wk | C, A | 6 | 17 | – |
| Paridaens *et al.*, 1985 | 100 mg m$^{-2}$ | q 4 wk | Vds | 46 | 19 | 5 |
| Cocconi *et al.*, 1986 | 80 mg m$^{-2}$ | q 3 wk | E | 30 | 17 | 4 |
| Tinsley *et al.*, 1986 | 20 mg m$^{-2}$ | q d × 5 q 3 wk | E | 42 | 17 | 2.5 |
| Gonzalez *et al.*, 1986 | 15 mg m$^{-2}$ | q d × 5 (frequency not given) | F | 16 | 68 | 6 + |
| Cox *et al.*, 1987 | 20 mg m$^{-2}$ | q d × 5 d q 3–6 wk | E | 11 | 37 | 6 |
| Zaniboni *et al.*, 1987 | 30 mg m$^{-2}$ | d 1,3,5 q 4 wk | C, Epi | 11 | 0 | – |
| Fornasiero *et al.*, 1987 | 30 mg m$^{-2}$ | d 1,3,5 q 3–4 wk | C, A | 45 | 45 | 7 |
| Cox *et al.*, 1989 | 20 mg m$^{-2}$ | q d × 5 q 3–6 wk | E | 29 | 38 | – |
| Hart *et al.*, 1989 | 20 mg m$^{-2}$ | q d × 5 q 4 wk | F, LV | 11 | 9 | – |
| Bitran *et al.*, 1990 | 100 mg m$^{-2}$ | q 4 wk | CiF | 24 | 50 | 4.9 |
| Krook *et al.*, 1990 | 45 mg m$^{-2}$ | ci q d × 2 q 4 wk | E | 44 | 25 | 4 |
| Saphner *et al.*, 1991 | 20–60 mg m$^{-2}$ | q wk × 7 | CiF, E | 13 | 15 | – |
| Khayat *et al.*, 1991 | 35 mg m$^{-2}$ | q d × 3 | E, CiF, A, M | 53 | 60 | – |
| Bromberg *et al.*, 1991 | 25 mg m$^{-2}$ | ci q d × 3 q 3 wk | E | 17 | 35 | 2 |
| Morere *et al.*, 1991 | 10 mg | iai q d × 6–13d q 4 wk | B + Vbl or M or F | 17 | 65 | – |
| Leong *et al.*, 1991 | 5–20 mg m$^{-2}$ | iv q d × 5 q 4 wk | F, LV | 19 | 42 | 2 |

Abbreviations: A = doxorubicin;. B = bleomycin; C = cyclophosphamide; ci = continuous infusion; E = etoposide; Epi = epirubicin; F = 5-fluorouracil; I = ifosfamide; iai = intraarterial infusion; LV = leucovorin; M = mitomycin-C; Vbl = vinblastine; Vds = vindesine.

cyclophosphamide, vinblastine, mitomycin C). Others include agents not frequently used for the treatment of this disease such as etoposide. The rationale for this approach is that the combination of cisplatin and etoposide has been shown to be active in other tumour types particularly small cell and non-small cell lung cancer. These studies are all uncontrolled, and often accrue only small numbers of patients. In many of the combinations, response rates are low, but it is worth noting that a few studies using cisplatin in combination with 5FU, with or without other additional agents, achieve response rates as high as 68% for second-line chemotherapy (Gonzalez *et al.*, 1986; Bitran *et al.*, 1990; Khayat *et al.*, 1991). This combination justifies further investigation.

*Cisplatin in conventional dose combination chemotherapy: first line treatment*

There is now a considerable literature on the role of cisplatin in combination chemotherapy in previously untreated patients and this is summarised in Table III. Again, some studies involve cisplatin in combination with conventional anti-breast cancer chemotherapy and others use the agent in combination with etoposide. Many of these studies are again uncontrolled and involve small numbers of patients. The overall trend suggests a higher response rate than for patients who have received previous chemotherapy.

Within this group there are four randomised trials comparing cisplatin combination chemotherapy with conventional regimens. In the first, 72 patients were randomised to receive either cyclophosphamide, doxorubicin and cisplatin (CAP) or cyclophosphamide, methotrexate, 5FU, vincristine and prednisolone (CMFVP) (Kolaric *et al.*, 1984). CAP achieved a significantly higher response rate of 75% compared with 44% ($P < 0.01$) for CMFVP but there was no significant difference in response duration or overall survival. Toxic side-effects were more pronounced with CAP, including in particular myelosuppression, anaemia and vomiting. In this trial 5/11 (45%) CMFVP-resistant patients showed a second-line objective response to CAP. In the second trial, a similar CAP protocol followed by maintenance cyclophosphamide, 5FU and prednisolone was compared with cyclophosphamide, 5FU and prednisolone along in a randomised trial of 86 patients of whom only seven had had prior chemotherapy (Creagen *et al.*, 1984). CFP (CFP) alone was associated with a response rate of 46%, a median time to progression of 9 months and a median survival of 18 months *vs* 49%, 6 months and 11 months respectively for CAP followed by CFP. In addition to this trend towards worse survival, the cisplatin combination was associated with a significant increase in nausea and vomiting. In the third trial, CAP achieved a response rate of 67% compared with 41% for the conventional FAC regimen (5FU, doxorubicin, cyclophosphamide), again without significant difference in median response duration or survival (Kolaric *et al.*, 1989). In the fourth trial, an Italian group compared a novel combination of cisplatin and etoposide with a standard CMF regimen (Cocconi *et al.*, 1991). Cisplatin and etoposide achieved a response rate of 63% compared with 48% for CMF ($P = 0.08$). There was no significant difference in time to treatment progression, response duration or survival, but haematological toxicity, nausea and vomiting were greater with the cisplatin/etoposide combination.

The overall impression from these trials is that first-line combination chemotherapy which included cisplatin may achieve slightly higher response rates than conventional schedules but with increased toxicity and without significant benefit in terms of response duration.

### *Cisplatin in high dose chemotherapy*

Cisplatin is hardly the ideal drug for dose escalation with autologous bone marrow rescue (ABMR) because of its important non-haematological toxicities. Nevertheless this agent has been used in several high dose combination schedules, summarised in Table IV.

In the first major study of its type, Eder *et al.* (1986) treated 17 patients with high dose cisplatin 165 mg m$^{-2}$, cyclophosphamide 5.625g m$^{-2}$ and BCNU 600 mg m$^{-2}$ with ABMR. Fourteen of 16 evaluable patients responded (88%), including six complete responders (38%). Thirteen of these patients had been previously treated. Median time to tumour progression and median survival were however disappoint-

**Table III** Combination cisplatin in previously untreated patients

| *Reference* | *Dose* | *Schedule* | *Concurrent treatment* | *No. eval.* | *Response rate (%)* | *Median response duration (mo)* |
|---|---|---|---|---|---|---|
| Mechl & Sopova, 1984 | 80 mg m$^{-2}$ | q 4 wk | C, A | 6 | 83 | 6.3 |
| Kolaric *et al.*, 1984 | 20 mg m$^{-2}$ | d 1,3,5, q 3–4 wk | C, A | 36 | 75 | 12+ |
| Creagen *et al.*, 1985 | 40 mg m$^{-2}$ | q 4 wk | C,A, F, P | 45 | 49 | 6 |
| Kolaric *et al.*, 1986 | 20 mg m$^{-2}$ | d 1,3,5 q 3–4 wk | C, A | 38 | 58 | 8+ |
| Zaniboni *et al.*, 1987 | 30 mg m$^{-2}$ | d 1,3,5 q 4 wk | C | 10 | 70 | 6.2 |
| Cocconi *et al.*, 1991 | 100 mg m$^{-2}$ | q 3 wk | E | 65 | 63 | 11 |
| Roth *et al.*, 1988 | 70 mg m$^{-2}$ | q 4 wk | Mtx, Vbl, A | 38 | 66 | 5+ |
| Verusio *et al.*, 1988 | 20 mg m$^{-2}$ | q d × 3 q 3 wk | C, E | 20 | 70 | 9 |
| Colozza *et al.*, 1989 | 20 mg m$^{-2}$ | d 1–3 q 3 wk | C, A | 33 | 64 | 11 |
| Kudelka *et al.*, 1989 | 70 mg m$^{-2}$ | q 4 wk | Mtx, Vbl, A, LV | 34 | 91 | – |
| Kolaric *et al.*, 1989 | 30 mg m$^{-2}$ | d 1,3,5 | C, A | | 67 | NS |
| Kolaric & Tomek, 1990 | 30 mg m$^{-2}$ | d 1,3,5 | C, Mtx, F, Vc, P, A, ('alternating CAP/CMFVP') | 45 | 82 | 12 |
| Langer *et al.*, 1991 | 70 mg m$^{-2}$ | q 4 wk | Mtx, Vbl, A | 29 | 86 | 5.5 |

Abbreviations: A = doxorubicin;. B = bleomycin; C = cyclophosphamide; ci = continuous infusion; E = etoposide; Epi = epirubicin; F = 5-fluorouracil; I = ifosfamide; iai = intraarterial infusion; LV = leucovorin; M = mitomycin-C; Mtx = methotrexate; P = prednisolone; Vbl = vinblastine; Vc = vincristine; Vds = vindesine.

**Table IV** High dose cisplatin with autologous bone marrow rescue

| *Reference* | *Previous chemotherapy for metastatic disease* | *Dose* | *Concurrent chemotherapy* | *Eval. Pts.* | *Overall response (%)* | *CR (%)* | *Median response duration (months)* | *Treatment-related deaths (%)* |
|---|---|---|---|---|---|---|---|---|
| Eder *et al.*, 1986 | 13 | 165 mg m$^{-2}$ | C 5.65 g m$^{-2}$ BCNU 600 mg m$^{-2}$ + ABMR | 16 | 88 | 38 | 5 | 18 |
| Peters *et al.*, 1988 | None | 165 mg m$^{-2}$ | C 6.65 mg m$^{-2}$ BCNU 600 mg m$^{-2}$ or Melphalan 40 mg m$^{-2}$ + ABMR | 22 | 77 | 54 | 9 | 23 |
| Peters *et al.*, 1990 | None | 165 mg m$^{-2}$ | C 5.6 mg m$^{-2}$ BCNU 600 mg m$^{-2}$ + ABMR | 35 | – (adjuvant) | | – | 11 |
| Jones *et al.*, 1990 | None | 55 mg m$^{-2}$ q d × 4 | AFMtx induction → C 1.875 mg m$^{-2}$ q d × 3 BCNU 600 mg m$^{-2}$ × 1 day + ABMR | 39 | 97 | 64 | – | 20 |
| Tenny *et al.*, 1990 | 10 | 40 mg m$^{-2}$ q d × 4 or Carboplatin 375 mg m$^{-2}$ q d × 4 | C 25–50 mg m$^{-2}$ q d × 4 E 375–560 mg m$^{-2}$ q d × 4 + ABMR | 7 | 100 | 43 | – | 43 |
| Huan *et al.*, 1991 | None | 120–165 mg m$^{-2}$ | Conventional chemotherapy → C 4.5–6 g m$^{-2}$ E 750–1500 mg m$^{-2}$ ± ABMR | 73 | 81 | 55 | – | not given |
| Gingrich *et al.*, 1991 | | 120–200 mg m$^{-2}$ | E 1600–2600 mg m$^{-2}$ C 160 mg m$^{-2}$ ± Thiotepa 180–480 mg m$^{-2}$ ± RT + ABMR | 52 | 37 | all CR | – | 10 |

E = etoposide; C = cyclophosphamide; AFMTX = doxorubicin, 5FU, methotrexate; ABMR = autologous bone marrow rescue.

ingly short at 5 months and 8 months respectively. There were three treatment-related deaths (18%) and causes of death included renal failure. Subsequently, Peters *et al.* (1988) at Duke University reported a similar study in which 22 premenopausal patients with oestrogen receptor negative disease were treated with an identical schedule except that melphalan 40 mg $m^{-2}$ was sometimes substituted for BCNU. In contrast to the first study, none of these patients had received previous chemotherapy for metastatic disease. Seventeen (77%) achieved a response including 12 (54%) complete responders. Median response duration was 9 months and median survival for all patients was 8 months. Three patients acheived unmaintained remission beyond 16 months. Five patients (23%) had treatment-related deaths. Other similarly designed studies so far involve patient numbers too small to draw meaningful conclusions (e.g. Tenny *et al.*, 1990).

A second approach with high dose chemotherapy is to use this as so-called consolidation after conventional induction treatment. The Duke University group have also used this approach with an induction schedule of doxorubicin, 5FU and methotrexate followed by intensive consolidation chemotherapy using cyclophosphamide 1.87g $m^{-2}$ daily × 3 days, cisplatin 55 mg $m^{-2}$ × 4 days and BCNU 600 mg $m^{-2}$ × 1 day (Jones *et al.*, 1990). This approach achieved a 97% response rate in 39 patients including 25 (64%) achieving a complete remission. Eventually, however eight patients (28%) died of treatment related toxicity. Using a similar approach, the MD Anderson group has very recently reported an overall response rate of 81% including 55% complete remissions using cyclophosphamide 4.5–6g $m^{-2}$, etoposide 750–1,500 mg $m^{-2}$ and cisplatin 120–165 mg $m^{-2}$ as consolidation following conventional induction chemotherapy (Huan *et al.*, 1991). However, a 74% objective response rate including 30% CR were achieved with conventional therapy alone. Mortality rate related to high dose therapy was not given.

Finally, in a provocative study, Peters *et al.* (1990) have reported preliminary results of high dose cisplatin as part of adjuvant chemotherapy. In this study high risk patients with early breast cancer and ten or more involved axillary nodes were treated initially with four cycles of conventional cyclophosphamide, doxorubicin and 5FU chemotherapy followed by high dose cisplatin 165 mg $m^{-2}$, cyclophosphamide 5.6g $m^{-2}$ and carmustine 600 mg $m^{-2}$ with ABMR. Four of 35 patients treated in this way died of treatment-related complications (11%); it is otherwise too early to draw conclusions from this study. A randomised comparative trial is now under way.

### Carboplatin

### *Single agent – previously treated patients*

Results with carboplatin in the treatment of advanced breast cancer follow early experience with cisplatin: clinical activity appears related to whether or not the patient has received previous chemotherapy.

In the earliest carboplatin study, carried out by CALGB, 20 patients were treated with a 24 h infusion of either 320 mg $m^{-2}$ if they were considered good risk, or 280 mg $m^{-2}$ if they were considered poor risk based on previous therapy with nitrosoureas, mitomycin-C or large volume radiotherapy (Booth *et al.*, 1985). Treatment was repeated every 28 days. All patients had been heavily pre-treated with conventional chemotherapy and had received a median of six previous drugs. Fourteen patients were evaluable for response, but no responses were seen.

More recently in a Spanish study, Martin *et al.* (1991) reported 14 previously treated evaluable patients given carboplatin in a dose of 400 mg $m^{-2}$ repeating 4 weekly. All but one of these had previously received a doxorubicin-containing regimen, usually FAC; eight of these had only received adjuvant chemotherapy. Again, no responses were seen.

We are currently carrying out a phase II study of single agent carboplatin in advanced breast cancer, using a pharmacokinetically determined dose related to renal function (Calvert *et al.*, 1989). Our aim is to achieve an area under the concentration *vs* time curve (AUC) of 7 mg $ml^{-1}$ $min^{-1}$. So far only one of eight previously treated patients have responded. Table V summarises these results and the overall response rate is only one out of 36 (3%).

### *Single agent – previously untreated patients*

Kolaric's group in Yugoslavia has recently followed up their original cisplatin work with a study using carboplatin in 20 patients who had received no previous chemotherapy (Kolaric & Vukas, 1990). This group attempted to give a dose of 400 mg $m^{-2}$ every 3 weeks, rather than every 4 weeks. All patients were evaluable; there were two CRs and two PRs giving a 20% overall response rate (95% confidence limits 6–44%). Remission durations ranged from 2–8 months with a median of 4 months. The increased frequency of scheduling was associated with a surprisingly modest degree of short-term myelo-suppression. Eight patients had leukopenia but only two grade 3/4; three patients had thrombocytopenia but only one was grade 3/4. Longer term myelosuppression was more of a problem however, and the maximum number of cycles that could be given was five. Seven out of 13 patients subsequently responded to conventional combination CMFVP chemotherapy (54%).

In the second part of the Spanish study mentioned above, 21 previously untreated patients were given carboplatin 400 mg $m^{-2}$ q 4 weekly (Martin *et al.*, 1991). Nineteen were evaluable for response and of these one achieved a CR and five a PR giving an overall response rate of 32% (13–57%). Response durations ranged from 5 to 15 + months. Only four patients were given six or more courses. Leukopenia and thrombocytopenia were mild. Five out of ten patients subsequently responded to conventional FAC chemotherapy including five out of eight failing to respond to carboplatin.

In a small joint Portugeuse-UK study reported by Carmo-Periera *et al.* (1990) only two of 15 previously untreated patients responded to carboplatin in a dose of 400 mg $m^{-2}$ every 4 weeks. Finally, in our own on-going pharmacokinetically determined study nine out of 21 previously untreated patients have so far responded.

These results are summarised in Table V. The overall response rate in previously untreated patients is 21/75 (28%). This suggests a lower response rate than for cisplatin, and if this is real then it is surprising; in other tumour types carboplatin appears to have broadly similar efficacy to cisplatin (Smith *et al.*, 1985; Wiltshaw *et al.*, 1985).

### *Carboplatin in combination chemotherapy*

There are relatively few published studies of carboplatin in conventional dosage as part of combination chemotherapy and these are listed in Table VI. In the majority of these carboplatin has been given with 5FU in patients who have already received prior chemotherapy, and response rates in small series range from 25–44% (Fernandez-Hidalgo *et al.*, 1989; Allegra, 1989; Khayat *et al.*, 1989). Carboplatin has also been used in an unconventional regimen with etoposide and ifosfamide, a combination that we have already found highly active in small cell lung cancer (Smith *et al.*, 1990). In a group of 26 breast cancer patients described as being refractory to chemotherapy a 42% response was achieved (Fields *et al.*, 1991). Meaningful conclusions about the role of carboplatin in combination chemotherapy cannot be drawn from the limited data in these studies.

### *Carboplatin in high dose combination chemotherapy with autologous bone marrow rescue*

Carboplatin is a much more appropriate drug than cisplatin for use in high dose chemotherapy studies with AMBR; its dose limiting toxicity is myelosuppression and we have found

**Table V** Carboplatin as single agent chemotherapy

| *Reference* | *Previous chemotherapy* | *Dose* | *Response* |
|---|---|---|---|
| Booth *et al.*, 1985 | Yes | 280–320 mg m$^{-2}$ q 4 wk | 0/14 |
| Martin *et al.*, 1991 | Yes | 400 mg m$^{-2}$ q 4 wk | 0/14 |
| O'Brien *et al.*, 1991 | Yes | AUC 7 mg ml$^{-1}$ min$^{-1}$ q 4 wk | 1/8 |
| | | Total | 1/36 (3%) |
| Kolaric & Vukas, 1990 | No | 400 mg m$^{-2}$ q 3 wk | 4/20 |
| Carmo-Periera *et al.*, 1989 | No | 400 mg m$^{-2}$ q 4 wk | 2/15 |
| Martin *et al.*, 1991 | No | 400 mg m$^{-2}$ q 4 wk | 6/19 |
| O'Brien *et al.*, 1991 | No | AUC 7 mg ml$^{-1}$ min$^{-1}$ q 4 wk | 9/21 |
| | | Total | 21/75 (28%) |

**Table VI** Carboplatin combination chemotherapy

| *Reference* | *Dose* | *Schedule* | *Concurrent treatment* | *No. eval.* | *Response rate (%)* | *Median response duration (mo)* |
|---|---|---|---|---|---|---|
| Field *et al.*, 1991 | 200 mg m$^{-2}$ | q d × 2 q 4 wk | I, E | 26 | 42 | – |
| Allegra *et al.*, 1989 | 50–100 mg m$^{-2}$ | q d × 3 q 4 wk | F, LV | 18 | 44 | 6.3 |
| Khayat *et al.*, 1989 | 350 mg m$^{-2}$ | ia q 4 wk | F | 4 | 25 | – |
| Fernandez-Hidalgo *et al.*, 1989 | 55 mg m$^{-2}$ | 3–5 d i.v. q 5 wk | F | 31 | 20 | – |

E = etoposide; F = 5FU; I = ifosfamide; LV = leucovorin.

that a 4-fold dose escalation to 1,600 mg m$^{-2}$ is clinically feasible (Gore *et al.*, 1987). The Boston group who pioneered high dose cisplatin chemotherapy have also carried out a similar study using dose-escalations of high dose carboplatin (400–1,000 mg m$^{-2}$) with cyclophosphamide 6G m$^{-2}$ and thiotepa 500–720 mg m$^{-2}$ (Eder *et al.*, 1990). Sixteen previously treated patients with metastatic breast cancer were included in this study of whom 13 (81%) responded including one CR. Twenty-seven patients altogether with different tumour types were entered; severe mucositis and neurotoxicity were dose-limiting and there were two treatment-related deaths (7%).

Other groups are now also substituting carboplatin for cisplatin (e.g. Tenny *et al.*, 1990) as part of high dose chemotherapy for programmes in the treatment of breast cancer. The problem here is the apparently lower response rate of the analogue compared with the parent compound. This highlights the need to find new cisplatin analogues with the activity of the parent compound and the toxicity spectrum of carboplatin.

### Iproplatin

Iproplatin is a second generation cisplatin derivative investigated in parallel with carboplatin. Its further development was curtailed by nephrotoxicity. During its period of clinical study, iproplatin was investigated by three separate groups in patients with advanced breast cancer, previously treated with chemotherapy (Meisner *et al.*, 1989; Casper *et al.*, 1988; Hortobagyi *et al.*, 1987). Only seven patients out of 83 responded (8%). Details are given in Table VII.

### Conclusions

Cisplatin has low activity as second-line treatment for advanced breast cancer but data from three small studies suggests that it is highly active as first-line treatment in maximum conventional dosage of 120 mg m$^{-2}$ every 3 weeks. It would be reassuring to have this confirmed in larger numbers of patients and it would also be helpful to have an indication of response rate at lower dosage. In practice, it is unlikely that such studies will be carried out. Cisplatin has also been shown to be active in combination chemotherapy but so far four randomised trials have failed to show survival benefit over conventional treatment and its toxicity makes it an awkward drug in this area of palliative medicine. It has been incorporated in several high dose chemotherapy regimens, but again its toxicity greatly limits its potential in this area.

Carboplatin has a toxicity profile that makes it much more appropriate for the treatment of breast cancer, both in conventional and in high dosage. Unfortunately, results so far suggest that its activity is lower than cisplatin in this disease, even in previously untreated patients. More data are required with carboplatin at higher dosage to justify its use in high dose combination chemotherapy.

Finally, results with cisplatin as front-line therapy suggest that breast cancer should be an important target tumour for new cisplatin analogues.

**Table VII** Iproplatin (CHIP): single agent treatment

| *Reference* | *Previous chemotherapy* | *Dose* | *Response* |
|---|---|---|---|
| Hortobagyi *et al.*, 1987 | Yes | 270–300 mg m$^{-2}$ q 3 wk | 4/30 |
| Casper *et al.*, 1988 | Yes | 275 mg m$^{-2}$ q 4 wk | 2/24 |
| Meisner *et al.*, 1989 | Yes | 45 mg m$^{-2}$ q d × 5 q 4 wk | 1/29 |
| | | Total | 7/83 (8%) |

### References

ALLEGRA, C.J., MAYER, A., REED, E. & 4 others (1989). Therapy of patients with metastatic breast cancer with 5-fluourouracil, leucovorin and carboplatin. *Proc. Am. Soc. Clin. Oncol.*, **8**, 207.

BAJORIN, D., BOSL, G.J. & FEIN, R. (1987). Phase I trial of escalating doses of cisplatin in hypertonic saline. *J. Clin. Oncol.*, **5**, 1589–1593.

BITRAN, J.D., KOZLOFF, M.F. & DESSER, R.K. (1990). Platinol (CDDP) and continuous intravenous infusion 5-fluorouracil in refractory stage IV breast cancer. A phase II study. *Cancer Invest.*, **8**, 335–338.

BOOTH, B.W., WEISS, R.B., KORZUN, A.H. & 3 others (1985). Phase II trial of carboplatin in advanced breast cancer carcinoma: a cancer and Leukaemia Group B study. *Cancer Treat. Rep.*, **69**, 919–920.

BROMBERG, C., REMICK, S., HARPER, G. & 6 others (1991). Concurrent 72 hours continuous infusion of etoposide and cisplatin in metastatic breast cancer. *Proc. Am. Soc. Clin. Oncol.*, **10**, 49.

CALVERT, A.H., HARLAND, S.J., NEWELL, D.R. & 9 others (1982). Early clinical studies with cis-diammine I,I-cyclobutane dicarboxylate platinum II. *Cancer Chemother. Pharmacol.*, **9**, 140–147.

CALVERT, A.H., NEWELL, D.R., GUMBRELL, L.A. & 7 others (1989). Carboplatin dosage: prospective evaluation of a simple formula based on renal function. *J. Clin. Oncol.*, **7**, 1748–1756.

CARMO-PEREIRA, J., OLIVERA COSTA, F., HENRIQUEZ, E. & 4 others (1989). Carboplatin as primary chemotherapy for disseminated breast carcinoma: a phase II study. 5th European Conference on Clinical Oncology, London, Abstr. P0971.

CASPER, E.S., SMART, T.C., HAKES, T.B. & 2 others (1988). Clinical trial of iproplatin (cis-dichloro-trans-dihydroxy-bis-isopropylamine platinum IV, CHIP) in patients with advanced breast cancer. *Invest. New Drugs*, **6**, 87–91.

COCCONI, C., TONATO, M., DICOSTANZO, F. & 4 others (1986). Platinum and etoposide in chemotherapy refractory breast cancer. A phase II trial of the Italian Oncology Group for Clinical Research. *Eur. J. Cancer Clin. Oncol.*, **22**, 761–764.

COCCONI, G., BISAGNI, G., BACCHI, M. & 9 others (1991). Cisplatin and etoposide as first-line chemotherapy for metastatic breast carcinoma: a prospective randomised trial of the Italian Oncology Group for Clinical Research. *J. Clin. Oncol.*, **9**, 664–669.

COLOZZA, M., GORI, S., MOSCONI, A.M. & 8 others (1989). Chemotherapy with cisplatin, doxorubicin and cyclophosphamide (CAP) in patients with metastatic breast cancer. *Am. J. Clin. Oncol.*, **12**, 137–141.

COX, E.B., BURTON, G.V., OLSEN, G.A. & 4 others (1987). Response of refractory breast carcinoma to a combination of cisplatin and etoposide. *Proc. Am. Soc. Clin. Oncol.*, **6**, 58.

COX, E.B., BURTON, G.V., OLSEN, G.A. & VUGRIN, D. (1989). Cisplatin and etoposide: an effective treatment for refractory breast carcinoma. *Am. J. Clin. Oncol.*, **12**, 53–56.

CREAGEN, E.T., GREEN, S.J., AHMANN, D.L. & 3 others (1984). A phase III clinical trial comparing the combination cyclosphosphamide, Adriamycin, cisplatin and cyclophosphamide, 5-fluourouracil, prednisone in patients with advanced breast cancer. *J. Clin. Oncol.*, **2**, 1260–1265.

EDER, J.P., ANTMAN, K., PETERS, W.P. & 5 others (1986). High dose combination alkylating agent chemotherapy with autologous bone marrow support for metastatic breast cancer. *J. Clin. Oncol.*, **4**, 2592–2597.

EDER, J.P., ELIAS, A., SHEA, T.C. & 8 others (1990). A phase I-II study of cyclophosphamide, thiotepa and carboplatin with autologous bone marrow transplantation in solid tumor patients. *J. Clin. Oncol.*, **8**, 1239–1245.

FERNANDEZ-HIDALGO, O., GIL, A., HENRIQUES, I. & 3 others (1989). Eficacia de carboplatino + 5 fluorouracilo en tumores solidos. *Oncologia*, **12**, 101–109.

FIELDS, K.K., SALEH, R.A., ZORSKY, P.E. & 5 others (1991). Treatment of refractory metastatic breast cancer with ifosfamide, carboplatin and etoposide. *Proc. Am. Soc. Clin. Oncol.*, **10**, 70.

FORASTIERE, A.A., HAKES, T.B., WITTES, J.T. & WITTES, R.E. (1982). Cisplatin in the treatment of metastatic breast cancer. A prospective randomized trial of two dosage schedules. *Am. J. Clin. Oncol.*, **5**, 243–247.

FORNASIERO, A., DANIELE, O., AVERSA, S.M.L. & 3 others (1987). A 5 day regimen of cyclo-phosphamide, Adriamycin, platinum (CAP) in refractory breast cancer. *Chemiotherapia*, **6**, 310–313.

GINGRICH, R.D., BURNS, L.J., WEN, B.C. & others (1991). A phase I/II study of high-dose chemotherapy with marrow stem cell support in advanced breast cancer. *Proc. Am. Soc. Clin. Oncol.*, **10**, 67.

GONZALEZ, F., ANTON-APARICIO, L., DY, C. & 5 others (1986). 120 hours simultaneous infusion cisplatinum and fluourouracil (5FU) in drug resistant metastatic breast cancer. *Proc. Am. Soc. Clin. Oncol.*, **5**, 59.

GORE, M.E., CALVERT, A.H. & SMITH, I.E. (1987). High dose carboplatin in the treatment of lung cancer and mesothelioma: a phase I dose escalation study. *Eur. J. Cancer Clin. Oncol.*, **23**, 1391–1397.

HART, L., CHUA, C. & BROPHY, L. (1989). Salvage chemotherapy for metastatic breast cancer using cisplatin, 5FU and leucovorin: a phase I-II study.

HORTOBAGYI, G.N., FRYE, D., HOLMES, F.A & 3 others (1987). Phase II trial of iproplatin in metastatic breast cancer. *Cancer Treat. Rep.*, **71**, 1193–1196.

HUAN, S., YAU, J., WALLERSTEIN, R. & 4 others (1991). Characteristics of long-term progression-free survivors after tandem high dose cyclophosphamide, etoposide and cisplatin for breast cancer patients. *Proc. Am. Soc. Clin. Oncol.*, **10**, 60.

JONES, R.B., SHPALL, E.J., ROSS, M. & 4 others (1990). AFM induction chemotherapy, followed by intensive alkylating agent consolidation with autologous bone marrow support for advanced breast cancer. Current results. *Proc. Am. Soc. Clin. Oncol.*, **9**, 30.

KHAYAT, D., BOREL, C., LECESNE, A. & 7 others (1989). Preliminary report of a pilot study on hepatic intra-arterial infusion (HIAI) of carboplatin (CBDCA) & 5FU in the treatment of hepatic metastases. *Proc. Am. Assoc. Cancer Res.*, **30**, 1083.

KHAYAT, D., BOREL, C.H., WEIL, M. & 3 others (1991). Promising preliminary results of a combination of cisplatin/etoposide/5U and alternatively adriamycin/mitomycin C in primary resistant breast cancer and in heavily pre-treated metastatic breast cancer. *Proc. Am. Soc. Clin. Oncol.*, **10**, 71.

KOLARIC, K. & ROTH, A. (1983). Phase II clinical trial of cis-dichlordiammine platinum (cis-DDP) for antitumorigenic activity in previously untreated patients with metastatic breast cancer. *Cancer Chemother. Pharmacol.*, **11**, 108–112.

KOLARIC, K., ROTH, A., VUKAS, D. & CERVEK, J. (1984). CAP (cyclophosphamide, adriamycin, platinum) vs CMFVP (cyclophosphamide, methotrexate, 5-fluorouracil, vincristine, prednisolone) combination chemotherapy in untreated metastatic breast cancer. *Cancer Chemother. Pharmacol.*, **13**, 142–144.

KOLARIC, K.I., VUKAS, D. & POTREBICA, V. (1986). CAP (cylophosphamide, adriamycin, platinum) *vs* FAC (5FU, adriamycin, cyclophosphamide) combination chemotherapy in untreated metastatic breast cancer: a preliminary report. *Proc. Am. Soc. Clin. Oncol.*, **5**, 77.

KOLARIC, K., VUKAS, D. & POTREBICA, V. (1989). Combination of cyclophosphamide, adriamycin and platinum (CAP) versus 5-FU, adriamycin and cyclo-phosphamide as primary treatment in metastatic breast cancer: results of a prospective randomised study. *Tumori*, **75**, 132–136.

KOLARIC, K. & TOMEK, R. (1990). Cisplatinum based alternating non-cross-resistant chemotherapy as a first-line treatment in metastatic breast cancer. A phase II study. *Tumori*, **76**, 472–475.

KOLARIC, K. & VUKAS, D. (1990). Carboplatin activity in untreated metastatic breast cancer – a phase II trial. *Proc. Am. Soc. Clin. Oncol.*, **9**, 26.

KROOK, J.E., LOPRINZI, C.L., SCHAID, D.J. & 6 others (1990). Evaluation of the continuous infusion of etoposide plus cisplatin in metastatic breast cancer. A collaborative North Central Cancer Treatment Group/Mayo Clinic phase II study. *Cancer*, **65**, 418–421.

KUDELKA, A., ABEL, W., BERKEN, A. & 4 others (1989). Phase II trial of methotrexate, vinblastine, doxorubicin, cisplatin and folinic acid in the treatment of locally advanced and metastatic breast cancer: an update. *Proc. Am. Soc. Clin. Oncol.*, **8**, 194.

LANGER, C.J., CATALANO, R., SAREN, B. & 2 others (1991). Efficacy of M-VAC in advanced measurable breast carcinoma: phase II pilot study. *Proc. Am. Soc. Clin. Oncol.*, **10**, 65.

LEONG, L., DOROSHOW, J., AKMAN, S. & 7 others (1991). Phase II trial of 5FU, folinic acid and cisplatinum in metastatic breast cancer. *Proc. Am. Soc. Clin. Oncol.*, **10**, 65.

MARTIN, M., DIAZ-RUBIO, E., CASADO, A. & VEGA, J.M.L. (1991). Phase II study of carboplatin in advanced breast cancer: preliminary results. *Semin. Oncol.*, **18**, 23–27.

MARTINO, S., SAMAL, B.A., SINGHAKOWINTA, A. & 4 others (1984). A phase II study of cis-diamminedichloroplatinum II for advanced breast cancer. Two dose schedules. *J. Cancer Res. Clin. Oncol.*, **108**, 354–356.

MECHL, Z. & SOPOVA, B. (1984). CAP (cyclophosphamide, Adriamycin, cisplatinum) in the treatment of advanced breast cancer. *Neoplasma*, **31**, 431–435.

MECHL, Z. (1988). Quoted as personal communication. In Sledge, G.W. & Roth, B.J. Cisplatin in the management of breast cancer. *Semin. Oncol.*, **16**, 110–115.

MEISNER, D.J., GINSBERG, S., DITCH, A. & 8 others (1989). A phase II trial of iproplatin (CHIP) in previously treated advanced breast cancer. *Am. J. Clin. Oncol.*, **12**, 129–131.

MORERE, J.F., BOAZIZ, C., BREAU, J.L. & 2 others (1991). Continuous intraarterial chemotherapy in locally recurrence breast cancer. *Proc. Am. Soc. Clin. Oncol.*, **10**, 50.

O'BRIEN, M.E.R., TALBOT, D.C., RAMAGE, F., THOMAS, M., AHERN, J. & SMITH, I.E. (1992). Carboplatin – has it a place in the treatment of breast cancer? *Br. J. Cancer*, **65**, Suppl XVI, 39.

OSTROW, S., EGORIN, M., AISNER, J. & 4 others (1980). High dose cis-diamminedichloroplatinum therapy in patients with advanced breast cancer: pharmacokinetics, toxicity and therapeutic efficacy. *Cancer Clin. Trials*, **3**, 23–27.

PARIDAENS, R., CLARYSSE, A., ROZENCWEIG, M. & 2 others (1985). Cisplatin plus vindesine in advanced breast cancer: a phase II trial of the EORTC breast cancer cooperative group. *Eur. J. Cancer Clin. Oncol.*, **21**, 595–599.

PETERS, W.P., SHPALL, E.J., JONES, R.B. & 4 others (1988). High-dose combination alkylating agents with bone marrow support as initial treatment for metastatic breast cancer. *J. Clin. Oncol.*, **6**, 1368–1376.

PETERS, W.P., SHPALL, E.J., JONES, R.B. & ROSS, M. (1990). High dose combination cyclo-phosphamide, cisplatin and carmustine with bone marrow support as initial treatment for metastatic breast cancer: three-six year follow-up. *Proc. Am. Soc. Clin. Oncol.*, **9**, 10.

ROTH, B.J., SLEDGE, G.W. Jr, WILLIAMS, S.D. & 2 others (1988). Methotrexate, vinblastine, doxorubicin and cisplatin as first-line chemotherapy in metastatic breast cancer patients. A phase II trial of the Hoosier Oncology Group. *Proc. Am. Soc. Clin. Oncol.*, **7**, 31.

SAPHNER, T., TORMEY, D.C., ALBERTINI, M. & WINOKUR, S. (1991). Phase I trial of continuous infusion 5FU with weekly bolus cisplatinum and etoposide. *Proc. Am. Soc. Clin. Oncol.*, **10**, 55.

SESSA, C., VERMORKEN, J. RENARD, J. & J 5 others (1988). Phase II study of iproplatin in advanced ovarian carcinoma. *J. Clin. Oncol.*, **6**, 98–105.

SLEDGE, G.W., LOEHRER, P.J., ROTH, B.J. & EINHORN, L.H. (1988). Cisplatin as first-line therapy for metastatic breast cancer. *J. Clin. Oncol.*, **6**, 1811–1814.

SLEDGE, G.W. & ROTH, B.J. (1989). Cisplatin in the management of breast cancer. *Semin. Oncol.*, **16**, 110–115.

SMITH, I.E., HARLAND, S.J., ROBINSON, B.A. & 4 others (1985). Carboplatin: a very active new cisplatin analog in the treatment of small-cell lung cancer. *Cancer Treat. Rep.*, **69**, 43–46.

SMITH, I.E., PERREN, T.J., ASHLEY, S.A. & 4 others (1990). Carboplatin etoposide and ifosfamide as intensive chemotherapy for small cell lung cancer. *J. Clin. Oncol.*, **8**, 899–905.

TENNY, C.M., JACOBS, S.A., STOLLER, R.G. & 2 others (1990). Ablative chemotherapy with cyclophosphamide, etoposide, cisplatin or carboplatin and autologous bone marrow rescue in patients with recurrent breast cancer. *Proc. Am. Soc. Clin. Oncol.*, **9**, 175.

TINSLEY, R., FUKS, J., KORZUN, A. & 4 others (1986). Cisplatin and etoposide for advanced breast cancer. A phase II trial. *Proc. Am. Soc. Clin. Oncol.*, **5**, 74.

VAN GLABBEKE, M., RENARD, J., PINEDO, H.M. & 6 others (1988). Iproplatin and carboplatin induced toxicities: overview of phase II clinical trial conducted by the EORTC early clinical trials cooperative group. *Eur. J. Cancer Clin. Oncol.*, **24**, 255–262.

VERUSIO, C., BAJETTA, E., FERRARI, L. & 3 others (1988). Cyclophosphamide, adriamycin and cis-platinum (CAP) in metastatic and locally advanced breast cancer. *Am. J. Clin. Oncol.*, **11**, 435–439.

WILTSHAW, E. (1985). Ovarian trials at the Royal Marsden. *Cancer Treat. Rev.*, **12**, 67–71. (Suppl.).

YAP, H.-Y., SALEM, P., HORTOBAGYI, G.N. & 4 others (1978). Phase II study of cis-dichlorodiammineplatinum (II) in advanced breast cancer. *Cancer Treat. Rep.*, **62**, 405–408.

ZANIBONI, A., MARPICATI, P., SIMOCINI, E. & 5 others (1987). Cyclophosphamide, epirubicin, and cisplatin (CEP) in advanced breast cancer: preliminary results. *Anticancer Res.*, **7**, 813–816.