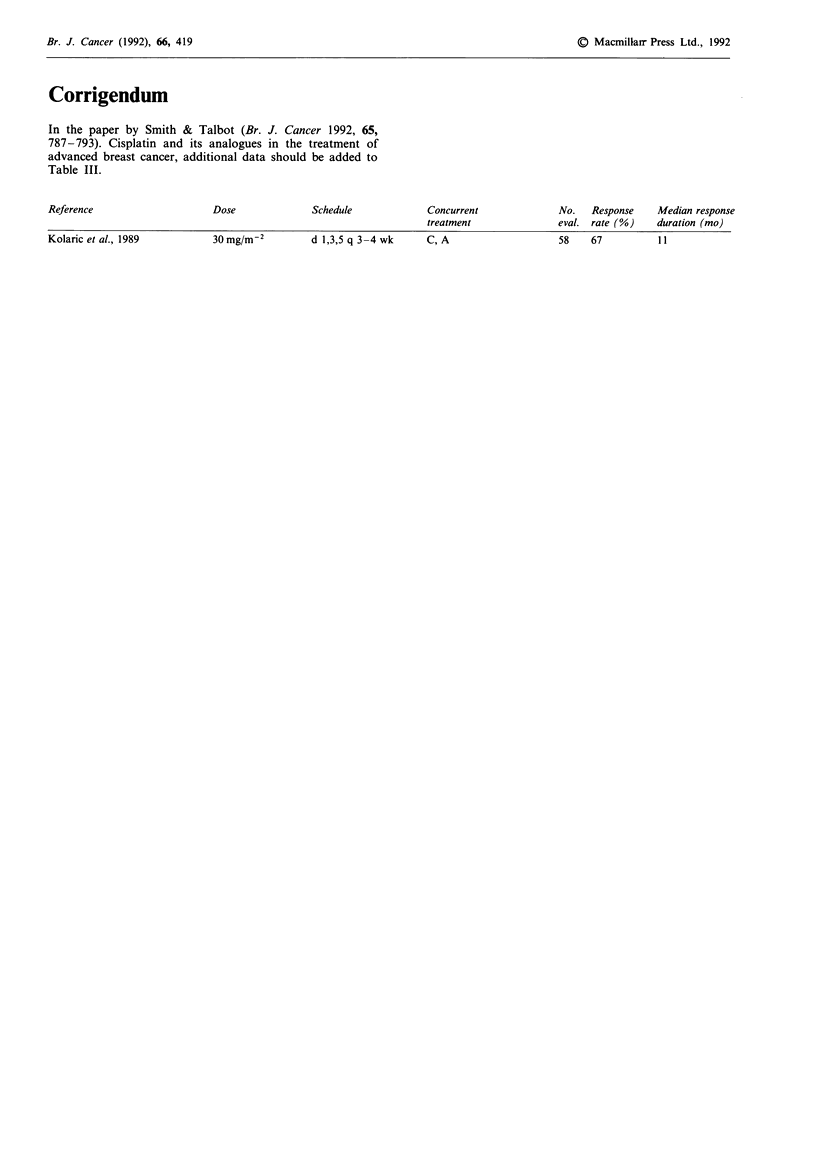# Corrigendum

**Published:** 1992-08

**Authors:** 


 © Macmillan Press Ltd., 1992

### Corrigendum

In the paper by Smith & Talbot (*Br. J. Cancer* 1992, **65**, 787–793). Cisplatin and its analogues in the treatment of advanced breast cancer, additional data should be added to Table III.

| *Reference* | *Dose* | *Schedule* | *Concurrent treatment* | *No. eval.* | *Response rate (%)* | *Median response duration (mo)* |
|---|---|---|---|---|---|---|
| Kolaric *et al.*, 1989 | 30 $mg/m^{-2}$ | d 1,3,5 q 3–4 wk | C, A | 58 | 67 | 11 |